# Nanometals-Containing Polymeric Membranes for Purification Processes

**DOI:** 10.3390/ma14030513

**Published:** 2021-01-21

**Authors:** Anna Rabajczyk, Maria Zielecka, Krzysztof Cygańczuk, Łukasz Pastuszka, Leszek Jurecki

**Affiliations:** Scientific and Research Center for Fire Protection National Research Institute, Nadwiślańska 213, 05-420 Józefów, Poland; mzielecka@cnbop.pl (M.Z.); kcyganczuk@cnbop.pl (K.C.); lpastuszka@cnbop.pl (Ł.P.); ljurecki@cnbop.pl (L.J.)

**Keywords:** nanoparticles of metals, polymeric membranes, modification, removal of impurities, aqueous solutions

## Abstract

A recent trend in the field of membrane research is the incorporation of nanoparticles into polymeric membranes, which could produce synergistic effects when using different types of materials. This paper discusses the effect of the introduction of different nanometals such as silver, iron, silica, aluminum, titanium, zinc, and copper and their oxides on the permeability, selectivity, hydrophilicity, conductivity, mechanical strength, thermal stability, and antiviral and antibacterial properties of polymeric membranes. The effects of nanoparticle physicochemical properties, type, size, and concentration on a membrane’s intrinsic properties such as pore morphology, porosity, pore size, hydrophilicity/hydrophobicity, membrane surface charge, and roughness are discussed, and the performance of nanocomposite membranes in terms of flux permeation, contaminant rejection, and antifouling capability are reviewed. The wide range of nanocomposite membrane applications including desalination and removal of various contaminants in water-treatment processes are discussed.

## 1. Introduction

In the last few decades, polymer membrane technology has become an efficient technique for water purification. A significant advantage of polymer membranes for wastewater treatment and clean-water production compared to conventional methods is the high purification capacity, ease of use, as well as cost-effectiveness. Polymeric membrane properties, including pore size, wettability, surface charge, roughness, thermal resistance, chemical stability, permeability, thickness, and mechanical strength, vary between membranes and applications. A recent trend in the field of membrane research is the incorporation of nanoparticles into polymeric membranes, which could produce synergistic effects when using different types of materials. Advanced nanocomposite membranes could be designed to meet specific water-treatment applications by tuning their structure and physicochemical properties (e.g., hydrophilicity, porosity, charge density, and thermal and mechanical stability) and by introducing unique functionalities (e.g., antibacterial, photocatalytic, or adsorptive capabilities). Advancements in membrane technology including new membrane materials, coatings, and manufacturing methods make it possible to obtain very good filtration and cleaning effects even in the case of mixtures that are difficult to separate. Polymer membranes are used for water treatment in industrial processes related to the production of food [1], textile [2], and petroleum products [3]. The removal of pollutants from drinking water is also a very important application [4,5,6,7].

In recent years, there has been significant progress in membrane processes, in which various solutions are used depending on the choice of filtering agent, which may be pressure, temperature, or an osmotic gradient [8,9]. The widely used pressure methods are classified according to pore size of the membrane: microfiltration, ultrafiltration, nanofiltration, and reverse osmosis [10]. A detailed overview of membrane technologies is presented in Table 1.

The main advantages of membrane processes are the production of pure water in one step and the possibility to use modular solutions, which allows easy scale-up. Accordingly, membrane processes can be used at all stages of a threat and at any level of intervention. The disadvantages of membrane processes are the deposition of contaminants on the membrane and the higher energy demand compared to conventional solutions. Currently, however, there are a number of innovative solutions that make it possible to obtain very good-quality permeate at reduced costs [17]. Constant progress in the field of membrane processes and the possibility of miniaturization of individual modules and scale-up depending on specific needs allows these processes to be more and more often used for water treatment in disaster areas. Membrane processes can also be effectively supported by combining various methods, e.g., photocatalysis/membrane processes [18,19]. A number of companies, including Norit, Berkfeld, and Kärcher, offer various solutions using these processes.

The aim of this review is to present the effect of introducing various metal nanoparticles, such as silver, iron, silica, aluminum, titanium, and other metals, and their oxides on various properties of polymer membranes, including selectivity, hydrophilicity, conductivity, mechanical strength, thermal stability, and antiviral and antibacterial properties, based on a literature review using the following keywords: polymer membranes, nanometals or metal nanoparticles in polymer membranes, modification of polymer membranes, and water and wastewater treatment. The literature review was performed on the following databases: Web of Knowledge, Scopus, Google Scholar Espacenet, Patentscope, and Google Patents. Some patents are also included in the description. Our review is divided into the following main sections: discussions on the effects of nanoparticle physicochemical properties, type, size, and concentration on membranes intrinsic properties such as pore morphology, porosity, pore size, hydrophilicity/hydrophobicity, membrane surface charge, and roughness, and on the performance of nanocomposite membranes in terms of flux permeation, contaminant rejection, and antifouling capability. The wide range of applications of nanocomposite membranes in water-treatment processes including desalination and removal of various contaminants are also discussed.

## 2. Membranes—Structure and Properties

The development of membrane techniques is closely related to research on new materials which extend the scope of separated mixtures. The aim is to improve the separation and transport parameters of membranes and to increase their durability. New materials should have high chemical resistance and temperature tolerance. Global solutions use ceramic, metallic, and carbon membranes, but the industry is still dominated by polymer membranes. The main drawback of inorganic membranes is a high cost, fragility, and stiffness, while polymer membranes are characterized by low stability at elevated temperatures and contamination. Profitability, good selectivity, and easy processing make polymer membranes more widely used, especially in water-treatment and desalination processes, and therefore, significant research and development efforts are being made to improve the essential parameters of polymer membranes [20]. The choice of material depends on the required parameters of the membranes used in various filtration methods and processes. Polymer membranes with differentiated porosities are mainly used in microfiltration (MF) and ultrafiltration (UF) and as pre-filtration membranes in nanofiltration (NF) and reverse osmosis (RO) [21].

Typically, polymer membranes are characterized by an integral structure consisting of an open porous support layer under a relatively thin, less porous skin layer of the same material. Separation takes place in the epidermal layer, while the support is easily permeable to water and non-separated substances dissolved in water. The highly selective surface layer of MF/UF membranes with pores from 0.01 to 0.2 mm is the active part of the membrane responsible for efficiency of the filtration process. Polymers such as polysulfone (PSF), polyethersulfone (PES), polyacrylonitrile (PAN), polypropylene (PP), polytetrafluoroethylene (PTFE), and polyvinylidene fluoride (PVDF) are the ones mainly used for the production of membranes for MF and UF. These materials are characterized by excellent permeability, selectivity, and stability (chemical, mechanical, and thermal) in water-treatment applications. The membrane materials primarily applied in UF, NF, and RO are PSF and PES [22]. 

Over the past decade, numerous trials have been devoted to manufacturing synthetic membranes for particular applications having appropriate features such as permeability, selectivity, and specific chemical and physical properties. To reach this target, various techniques have been performed such as phase inversion, track-etching, stretching, sintering, dip-coating, electrospinning and interfacial polymerization, and template leaching [23].

**Figure 1:**
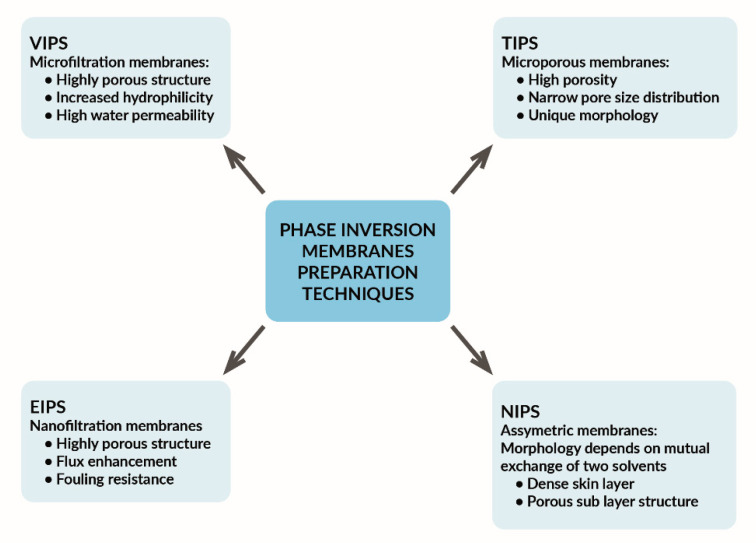
The effects of applied phase inversion techniques on the membrane properties.

The phase inversion method is the most important and common membrane fabrication process for both laboratory and commercial membranes. Phase inversion is defined as a de-mixing process in which a homogeneous liquid polymer solution is converted to a solid (using a coagulation bath) in a controlled manner by replacing the solvent from the polymer solution with a non-solvent in a coagulation bath [24]. The phase inversion technique is used to prepare an asymmetric membrane with a dense and thin layer of epidermis. The choice of solvent, polymer solution composition, solvent free system, casting conditions, and coagulation bath composition are some of the key factors that influence the phase inversion method in membrane formation [25]. The phase inversion method is divided into four different types: Non-Solvent-Induced Phase Inversion (NIPS), Thermally Induced Phase Inversion (TIPS), Evaporation-Induced Phase Inversion (EIPS), and Vapor-Induced Phase Inversion (VIPS); see also Figure 1.

The use of the Thermal-Induced Phase Inversion (TIPS) and Non-Solvent-Induced Phase Inversion (NIPS) techniques enables the preparation of a membrane fabrication with a desired morphology [26]. Comparing the TIPS and NIPS techniques, it should be emphasized that the use of TIPS allows for obtaining membranes with better parameters, especially in the field of permeation performances, mechanical strength, and pore size distribution [27,28]. TIPS can be used to make membranes from various polymers such as polyethylene, polyacrylonitrile, polypropylene, poly(vinylidene fluoride), and poly(methyl methacrylate) [29,30].

Most industrial membranes are prepared with NIPS or TIPS probably because VIPS and EIPS run slower than NIPS and TIPS and therefore allow a longer period of phase separation [31]. VIPS shows strong similarity to the NIPS method, while VIPS gives the possibility of better control of the membrane morphology during phase separation and has great potential for use in solutions used in water- and wastewater-treatment processes (MF, UF, NF, and Direct contact membrane distillation (DCMD)). However, it should be remembered that processes such as aggregation of the polymer may occur during the scale-up of production and that, in VIPS, the influx of a non-solvent is responsible for phase separation while, in EIPS, the efflux of solvent is responsible for phase inversion [32].

Despite continuous improvement in membrane manufacturing techniques, there is a need to optimize and enhance the separation performance of these polymeric membranes [3] as well as to improve some other physical properties such as stability, hydrophilicity profile, and fouling resistance [33].

In water systems, the biggest problem is membrane fouling, which is the most important limitation in their larger-scale use [34]. Biofouling is a consequence of the irreversible adhesion of microbial cells of one or more types of bacteria followed by colonization of the membrane surface, forming a microbial biofilm [35]. In addition, the deposition of natural organic matter and inorganic compounds on the membrane surface and inside the pores is an additional obstacle leading to membrane contamination. A biofilm hinders penetration of the solvent through the membrane, which leads to an increase in the transmembrane pressure necessary to achieve the assumed efficiency of the filtration process. Removal of the biofilm formed on the membrane surface is usually extremely difficult even with the use of biocides [36]. Cleaning the membranes from the deposited biofilm consumes a large amount of cleaning agents that can damage the membrane surface. Moreover, the necessity to clean the membrane significantly increases the operating costs of the sewage treatment plant [37].

The phenomenon of membrane fouling has been studied in many directions in order to understand the mechanisms and to determine the factors and types of sediments that affect fouling. Unfortunately, various methods of modifying the surface of the membranes aimed at reducing the deposition of fouling, for example, by grafting hydrophilic compounds onto the membrane, have not been satisfactory. Only the use of additives and processes in the field of nanotechnology make it possible to obtain membranes meeting the requirements of water-treatment processes. Developed in recent years, polymer nanocomposite membranes, also referred to as the nanocomposite mixed matrix membranes (MMNM), are advanced solutions in materials obtained primarily through the use of nanocomposites comprising nanoparticles dispersed in a polymer matrix [38,39]. The introduction of nanomaterials to the structure of polymer membranes allows for significant improvement and adaptation to the needs of a given application, such as hydrophilicity; charge density; porosity; and chemical, thermal, and mechanical stability [40,41]. In addition, significant improvements in physical properties such as strength and modulus can be achieved through strong interfacial interactions between the nanoparticles and the surrounding polymer matrix [42]. Due to the appropriate selection of metal nanoparticles or their oxides, unique functions such as antibacterial and photocatalytic properties can also be obtained. Modern polymer nanocomposite membranes can be used for various membrane processes, such as gas–gas, liquid–liquid, and liquid–solid separation [43]. One of the first effective polymer nanocomposite membranes was membranes obtained by introducing selective zeolite, thanks to which both permeability and selectivity were increased and which was used in gas separation [44,45]. These membranes have been successfully used in various other solutions such as proton exchange membrane fuel cells (PEMFC) [46,47], methanol fuel cells [48], lithium-ion batteries [49], sensors [50], pervaporation (PV), [51] and organic solvent nanofiltration (OSN) [52] as well as in water treatment.

**Figure 2:**
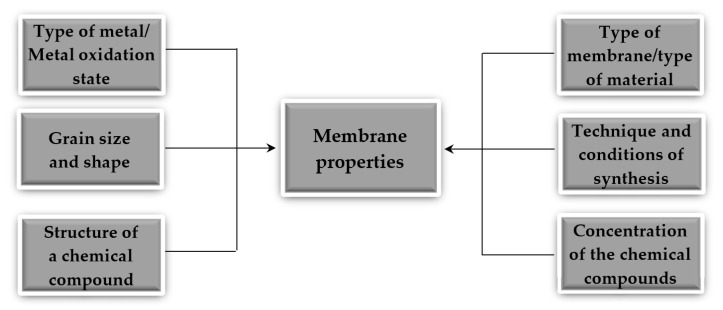
The main factors influencing the nature of the material of the membrane and its properties.

The most intensively studied direction in this regard is the introduction of different nanoparticles including nanoparticles of metals such as silver, iron, copper, zirconium, silica, aluminum, titanium, and magnesium and their oxides. The nanoparticles can be applied to the membrane surface or dispersed in a polymer solution prior to casting the membrane [53]. The method and conditions of synthesis, the nanoparticles used together with the type of metal, and their physicochemical characteristics determine the nature of the material and its properties (Figure 2).

The key factor influencing the quality of the polymer nanocomposite membrane is a very good dispersion of metal nanoparticles or their oxides in the membrane structure. The method of producing the membrane must provide adequate control of the aggregation/dispersion behavior. Aggregation can be caused by a series of surface interactions such as Van der Waals interactions, overlap of electric double layer, steric interaction of adsorbed polymer, and bridge or hydration forces [54,55]. Therefore, the first stage, to a large extent, determining the possibility of dispersing nanoparticles in the polymer matrix is the preparation of a homogeneous solution of polymer and particles. The following three methods of obtaining such a homogeneous solution can be distinguished:

- Nanoparticles are dispersed in the solvent and stirred for a period of time needed to obtain a homogeneous suspension, and then, the polymer is added [56,57].
- The polymer is dissolved in the solvent and stirred for a period of time needed to obtain a homogeneous polymeric solution, and then, nanoparticles are added [58,59].
- Nanoparticles are dispersed in the solvent and stirred, and the polymer is dissolved in a solvent separately; the nanoparticle suspension is then added to the homogeneous polymeric solution [60].

Of these methods, the first and third are more suitable for dispersing inorganic particles in a slurry because of the low viscosity of the starting filler/solvent slurry. Moreover, in the diluted suspension, the high shear rate during mixing prevents agglomeration of the particles [61]. 

In order to prepare the new modified membrane, the obtained polymer/nanoparticles homogeneous suspension should be further processed according to the methods used for the preparation of polymer membranes. Phase inversion [62], stretching [63], track-etching [64], and electrospinning [65] are the most commonly used to fabricate a membrane from a given material [62,66,67]. The choice of method depends on the planned properties and structure of the membrane and the type of homogeneous suspension. The most universal method and the most frequently used is the phase inversion described earlier.

## 3. Membranes Containing Nanoparticles of Silver

Silver (Ag) is the most widely studied antimicrobial agent in nanocomposite membranes due to its excellent biocidal properties. Agents with the participation of silver and its compounds show biocidal activity, but the mechanism of their cytotoxic activity is not fully elucidated. The sensitivity of microorganisms depends on the form of the introduced particles, either ionic or molecular [68,69,70]. There are a number of reports that the biocidal effectiveness of silver ions is lower compared to silver nanoparticles (AgNPs) showing particularly active inhibitory and antimicrobial properties and a broad spectrum of biocidal activity [71,72,73]. However, the mechanism of silver’s antibacterial action is still not fully understood. It is commonly believed that the interaction of silver with thiol groups [74,75] plays a major role in the degradation of bacteria. Moreover, when AgNPs are small enough to disrupt bacterial cell membranes, they can enter and disrupt the function of cellular enzymes [76,77]. The resistance of bacteria to silver also depends on the structure of their cell walls. AgNPs inactivate bacteria by stimulating dysfunction of their cell walls, e.g., by increasing its permeability [61]. An essential role in the intercellular transport of AgNPs is played by the endocytosis process, which consists in transporting particles together with a fragment of the cell membrane. As a result, the ability to replicate DNA is lost [78] and enzymes responsible for the proper functioning of many metabolic pathways and the respiration process are inactivated [61]. Disturbances of the structure and of the cell function lead to deactivation of the biochemical processes taking place in it.

An important problem hindering the effective use of AgNPs is their high agglomeration ability, which can result in a decrease in their antimicrobial and antifungal properties. A number of methods have been developed to facilitate good dispersion and stabilization of dispersed AgNPs in the polymer matrix. The use of a mixture of polyvinyl-pyrrolidone or 2,4,6-triaminopyrimidine [79,80] was used as a dispersant or compatibilizer in a polymer casting solution to facilitate the dispersion of AgNPs, which allowed an improvement in antibacterial properties. Good results were also obtained by applying a pretreatment of AgNPs with amphiphilic polymer in order to obtain better dispersion and compatibility of AgNPs with a polymer matrix [81].

Another problem that hinders the use of AgNPs is the susceptibility of these nanoparticles to leaching from the polymer matrix forming the membrane. AgNPs can be eluted either as metal nanoparticles or in dissolved form as Ag+ ions, which in both cases reduces the antimicrobial ability of membranes. Methods were developed to prevent leaching by incorporating silver embedded in the zeolite [82] or by grafting them onto the membrane surface through stronger bonds (e.g., electrostatic attraction or chemical bonding) [83]. 

The other effective method of preventing agglomeration and elution of AgNPs from the polymer matrix is the immobilization of AgNPs formed by an in situ method on the surface of sol-gel-derived silica nanoparticles containing reactive silanol groups enabling permanent incorporation of silica particles with immobilized AgNPs into the structure of the polymer matrix [84,85].

Despite the difficulties described above, the addition of AgNPs is widely used. Thanks to the introduction of AgNPs into the polymer membrane based on polysulfone for ultrafiltration, improved biofouling resistance and virus removal were obtained [86]. AgNPs incorporated into polysulfone ultrafiltration membranes exhibited antimicrobial properties towards a variety of bacteria, including *Escherichia coli* K12 and *Pseudomonas mendocina* KR1, and the MS2 bacteriophage. Nanoparticles of silver incorporation also increased the membrane hydrophilicity, reducing the potential for other types of membrane fouling. X-ray Photoelectron Spectroscopy (XPS) analysis indicated a significant loss of silver from the membrane surface after a relatively short filtration period (0.4 L/cm^2^) even though an Inductively Coupled Plasma (ICP) analysis of the digested membrane material showed that 90% of the added silver remained in the membrane. This silver loss resulted in a significant loss in antibacterial and antiviral activity. Thus, successful fabrication of AgNP-impregnated membranes needs to allow for the release of sufficient silver ions for microbial control while preventing rapid depletion of silver. The introduction of biogenic silver metallic nanoparticles into polyethersulfone (PES)-based membranes significantly reduced the biofouling of these membranes [87]. The results demonstrated that AgNPs were uniformly distributed on a membrane surface. Bio-Ag0 incorporation slightly increased the hydrophilicity of the PES membrane and increased the permeate flux. The antibacterial and anti-biofouling properties of the bio-Ag0/PES nanocomposite membrane were tested with pure cultures (*Escherichia coli* and *Pseudomonas aeruginosa*) and a mixed culture (an activated sludge bioreactor), respectively. The bio-Ag0/PES composite membranes, even with the lowest content of biogenic silver (140 mg bio-Ag0 m^−2^), not only exhibited excellent antibacterial activity but also prevented bacterial attachment to the membrane surface and decreased the biofilm formation during a 9-week test. 

In addition to the biofouling effect, AgNPs may have a catalyst facilitating the simultaneous separation and catalysis of organic pollutants. Very good long-term stability of the membrane properties was obtained by introducing AgNPs within inner pores of the ultrafiltration membrane based on PES and PES/TA fabricated by the non-solvent-induced phase separation method [88]. AgNPs were formed by a facile in situ blending and reduction method by reduction on the natural polyphenol tannic acid (TA)–Fe complex, which was firstly blended in a PES ultrafiltration membrane. The evenly distributed TA provides a convenient platform for forming and immobilizing catalytic AgNPs on the membrane matrix. Thanks to this feature, most of the AgNPs were distributed on the surface of inner pores and protected with a membrane separation layer from the macromolecular pollutants. The efficiency of the ultrafiltration process when using the described membrane was assessed on the basis of the measurements of pure water permeability, bovine serum albumin concentration (BSA), and humic acid decomposition (HA). It was found that the use of a membrane allows to obtain a continuous stream of pure water (239.8 L/m^2^·h), increased BSA separation (96.1%), and excellent HA separation (87.3%). Catalytic performance was evaluated by the reduction reaction of 4-nitrophenol (4-NP) as a target impurity. The 4-NP conversion was 98.0% for solution catalysis of the mixture containing HA and 4-NP in the dynamic mode compared to 55.8% in the static mode. No negative influence of HA on the catalytic activity of AgNPs was observed due to the distribution of these nanoparticles in the internal pores. The membranes produced were found to maintain a conversion factor greater than 95.0% for filtering the mixture solution for seven cycles. The obtained results indicate a high potential of these membranes for continuous reduction of 4-NP as well as for effective separation of high-molecular impurities. Despite the difficulties associated with agglomeration and elution of AgNPs, with correct introduction and immobilization in the polymer matrix, it is possible to obtain a number of favorable membrane properties and, in particular, to overcome biofouling [88]. Detailed examples of the use of different types of silver in nanomembranes are presented in Table 2.

## 4. Membranes Containing Nanoparticles of Silica

In many industrial filtration processes, good mechanical properties of the membrane are required. The incorporation of silica into a polymer composite is a known and widely used method to improve the mechanical properties and thermal stability of polymer composites. Based on an evaluation of the mechanical strength of mixed-matrix membranes based on polysulfone containing inorganic fillers such as silver, copper, silica, zeolite, and silver-zeolite, it was found that good dispersion of these fillers allows for increased hydrophilicity of the membrane and improvement of mechanical strength in terms of tensile strength and break elongation [95]. It is particularly important to obtain an improvement in the properties of the membrane to change the structure of the filler/polymer composite to replace the disordered with sponge-like macrovoids. As we know, silica is used in various forms, but in the composites used for the production of membranes, nanosilicas are most often used, including functionalized, mesoporous silicas, and polysilsesquioxanes (POSS) characterized by a designed spatial structure and a specific content of functional groups [96]. Good filler dispersion and the content of functional groups capable of reacting with the functional groups of the polymer enable permanent incorporation into the structure of the polymer nanocomposite from which the membrane is made. Detailed examples of the use of different types of silicas in nanomembranes are presented in Table 3.

From the data presented in Table 3, it can be seen that the addition of various types of silicas affects properties such as water filtration rate, thermal stability, separation performance, and hydrophilicity. The properties related to the filtration efficiency of aqueous mixtures largely depend on the hydrophilicity of the membrane [104]. Water flux was determined to depend mainly upon the IP film thickness and surface hydrophilicity, whereby these two parameters have counterbalancing effects. An increase in the hydrophilicity of membranes could facilitate water solubilization and diffusion through the membrane, thus improving water permeability. Almost all the studies using hydrophilic nanofillers resulted in a membrane with decreased contact angle, indicating an enhanced surface hydrophilicity. For example, for the mesoporous silica-PA membranes, the contact angle was decreased from around 57° to 28° with increasing silica loading from 0 to 0.1% (w/w) in the organic phase [100]. All examples presented in Table 3 indicate an enhanced water permeability compared to the parameters for membranes without silica-based fillers. Apart from changes and ordering of the membrane structure, an additional reason may be the fact that the embedded hydrophilic nanomaterials can be exposed on the membrane surface, providing more hydrophilic functional groups to membrane surface.

## 5. Membranes Containing Nanoparticles of Aluminum

Nanoparticles of Al_2_O_3_, Al, or in combination with other metals are most often used to modify membranes. The modifications depend on the purpose of the modified material. Nanometric alumina particles, compared to their micrometric counterparts, are characterized by a lower melting point, increased light absorption, better dispersion in both aqueous and inorganic solvents, and a much larger specific surface [105]. Aluminum nanoparticles, thanks to their structure, i.e., pore volume and size, degree of crystallinity, phase composition, and surface composition, are very good catalysts. In the case of aluminum oxide nanoparticles, we are dealing with substances that occur in various crystalline phases; show a strong ionic interatomic bond; and are resistant to acids and bases, even at elevated temperatures. In addition, it is characterized by high thermal conductivity, low electrical conductivity, and catalytic properties, especially γ-Al_2_O_3_, which has a large surface area and a large volume of open mesoporosity, conditioning rapid and even penetration into catalytic sites. On the other hand, α-Al_2_O_3_ is characterized by high strength, fire resistance, as well as anti-friction and insulating properties [106]. 

Aluminum nanocomposites, such as Al_2_S_3_, AlN, and others, are also characterized by specific properties that, introduced into the membrane structure, can significantly determine the parameters of the membrane and can influence environmental processes. Aluminum nanoparticles are introduced into membranes used in solution purification processes. However, it should be remembered that an optimal amount of nanoparticles is necessary as an excessive addition may lead to a decrease in the strength of the membrane [56]. An example of the importance of the amount of nanoparticles introduced to modify the material is the work of Yan et al. [107]. Polyvinylidene fluoride (PVDF) is a material that can form asymmetric membranes. This material makes it possible to produce membranes with high surface permeability and porosity, and good pore structure, being thermally stable and resistant to corrosion against most chemicals and organic compounds. In addition, PVDF membranes are characterized by permanent antioxidant activity, high hydrolytic stability, and good mechanical properties, which is why PVDF membranes are used in ultrafiltration processes. However, thanks to modifications, including the introduction of Al_2_O_3_ or Al nanoparticles, the possibilities and scope of their application have definitely increased [107]. Due to the introduction of Al_2_O_3_ nanoparticles, PVDF membranes improved the tensile strength parameters and elongation at break by more than 50%, however, only when the concentration of Al_2_O_3_-NPs was at maximum, 2% (by weight). The introduction of more Al_2_O_3_ nanoparticles caused the membrane flexibility to decrease. For the membrane containing 2% (by weight) of Al_2_O_3_-NPs, the hydrophilicity of the composite membrane, the antifouling effect and the flux drop rate were improved, which was only 18.2% at a transmembrane pressure of 0.1 MPa. However, the addition of Al_2_O_3_ nanoparticles did not affect the pore size, number of pores, or the formation of PVDF membrane crystals [107]. 

Membranes used in bioreactors (MBR) are an important tool in water- and wastewater-treatment systems. However, due to their hydrophobic nature, many of them face the problem of susceptibility to contamination [108]. Therefore, the conducted research focuses not only on improving the efficiency but also on reducing costs related to maintenance and operation of the installation. Maximous et al. [109] carried out work to obtain Al_2_O_3_-entrapped polyethersulfone (PES) ultrafiltration membranes. The modified membrane was applied to activated sludge filtration. The addition of Al_2_O_3_ nanoparticles made it possible to change the membrane efficiency, to lower the flux drop compared to a pure polymer membrane, to increase the porosity, and to reduce a hydrophobic interaction between the membrane surface and impurities [109]. 

Many studies have shown the need to introduce an appropriate amount of Al_2_O_3_ nanoparticles, i.e., adequately to the material undergoing modification (Table 4) and the purpose of the modifications.

The introduction of Al_2_O_3_ nanoparticles may influence the increase in the water stream continuously; however, it is not the same as the increase in the degree of reduction of the pollutant concentration. This behavior is attributed to enhancing the hydrophilicity and to increasing either the surface roughness or the porosity of the membrane [116]. However, it should be emphasized that the results of many studies confirm the need to select an appropriate amount of nano-Al_2_O_3_. Li et al. [117] have shown, for example, that increasing the number of atomic layer deposition (ALD) cycles to deposit Al_2_O_3_ nanoparticles on a polycarbonate (PCTE) membrane will result in a reduction of the water stream due to a reduction of the mean pore size as the Al_2_O_3_ layer thickens [117]. Therefore, when modifying the selected membrane material, it is necessary to determine the appropriate dose of n-Al_2_O_3_. Only then is it possible to effectively use the membrane in the processes of wastewater treatment and removal of pollutants from water.

Sequential Infiltration Synthesis (SIS) shows some similarity to atomic layer deposition (ALD) but differs qualitatively from ALD. SIS is where polymers are introduced into inorganic materials with alternating exposure to two chemical vapors. However, the use of this technique has limitations. SIS cannot be used with nonpolar polymers such as polystyrene (PS) which do not have functional groups that can bind to precursor species [118,119,120]. Waldman et al. [119] as well as Bergsman et al. [120] used sequential infiltration synthesis of trimethylaluminum to modify polyethersulfone, which is widely used in membrane filtration. As a consequence of the conducted process, the PES completely loses its original porosity, while the sample infiltrated with alumina remains mostly porous with little deformation. The presence of alumina changes the rheological properties of SIS-PES membranes and stabilizes the membrane structure at elevated temperatures. 

## 6. Membranes Containing Nanoparticles of Titanium

Titanium oxide nanoparticles are most often used in environmental engineering and are known for their photocatalytic properties, resulting directly from its method of processing, which determines the crystal structure. Nanoparticles of TiO_2_ are stable compounds, insoluble in water and diluted with acids or ordinary organic solvents, which is important for its applicability. Moreover, thanks to the high dielectric constant, it has very good electrical properties. It is a large band semiconductor; however, n-TiO_2_ in the anatase form shows higher photoactivity than other types of n-TiO_2_ [121]. It should be noted that the effectiveness of the introduced modifications with TiO_2_ nanoparticles depends on the size of the nanoparticles, the type of modified material, and the applied membrane production procedures. One study on the flow of water through a polyethersulfone-n-TiO_2_ membrane indicated the dependence of the flux effect on the concentration of nanoparticles, while other studies did not confirm this relationship [108]. The catalytic properties of TiO_2_ nanoparticles may increase the resistance to fouling and water flow, leading to the decomposition of organic compounds, which is why they are increasingly used in the modification of polymer membranes [116,122]. 

One of the most widely used membrane materials is polyvinylidene fluoride, which is internally hydrophobic in nature. Modification of the PVDF membrane with TiO_2_ nanoparticles was carried out, among others, by Wang et al. [123]. TiO_2_ was applied to PVDF ultrafiltration membranes using an atomic layer deposition technique with TiCl_4_ and water as the precursors. The membrane PVDF modified by n-TiO_2_ was characterized by increased hydrophilicity and hence better resistance to protein contamination. The membrane showed better properties in terms of hydrophilicity and fouling resistance with a greater number of n-TiO_2_ coating cycles, which decreased with more than 120 cycles [123].

One example of membrane modification with nanoparticles of TiO_2_ is the modification of a poly(vinylidene fluoride) (PVDF)/sulfonated polyethersulfone (SPES) membrane. TiO_2_ nanoparticles caused the average pore size of the membrane in the surface and bottom layer to decrease, while the hydrophilicity was increased. The antifouling properties of the membranes were improved by changing the membrane surface from hydrophobic to hydrophilic. The conducted studies also showed a significant photo-bactericidal effect in relation to *Escherichia coli* [124]. Bactericidal properties are important for wastewater-treatment processes where disinfection and removal of microorganisms is one of the important processes.

Modification of the membranes is also carried out in order to create systems targeted at specific pollutants, including, e.g., removal of organic pollutants. An example is a thin-layer nanocomposite (TFN) nanofiltration membrane created using a copolyamide layer as the solvent-stable membrane. The thin layer was modified by reacting TiO_2_ nanoparticles with a particle size of 25 nm in situ with the formation of a polyamide copolymer network on a polyimide (PI) support. Additionally, amine and chloride compounds were used to improve the compatibility of nanoparticles inside the polymer matrix. The conducted experiments in the field of transport properties of membranes have shown that the modified membranes are characterized by a higher methanol flow and good dye rejection despite a lower degree of swelling. Moreover, the type of chemical function, the n-TiO_2_ loading, and the pore size of the membrane have been identified as key factors influencing its performance [125].

Membranes modified by introducing TiO_2_ nanoparticles are primarily used to reduce pollution caused by organic matter. Composite PES/n-TiO_2_ membranes were prepared by the phase inversion method. TiO_2_ addition at 0.5 wt% enhanced properties such as hydrophilicity, thermal stability, mechanical strength, and antifouling capacity of the membrane without significantly changing the structure of the membrane. However, an n-TiO_2_ content greater than 0.5 wt% resulted in a defective pore structure of the membranes and a decrease in parameters such as permeability and mechanical strength. The membrane developed in this way may allow for more efficient purification processes due to the better antifouling properties of the membrane compared to membranes without TiO_2_ nanoparticles [126].

Similarly, studies by Luo et al. [127] show that the amount of TiO_2_ nanoparticles is important for effective operation of the membrane. They modified PES membranes with n-TiO_2_ using the sol-gel method; 5, 10, and 15 wt% were used for modification. For TiO_2_ nanoparticles, however, the most optimal PES/n-TiO_2_ properties were obtained for a composition containing less than 15 wt% nanoparticles of TiO_2_ [127]. Synthesis of the TiO_2_/3-cyanopropyltriethoxysilane (CPTES)/metformin-polyethersulfone (PES) membrane with various NP doses (0.1%, 0.5%, and 1% by weight) allowed to obtain a material with high antifouling properties. Operation of the membranes was tested by the removal of Cu(II) ions, the content of the chemical oxygen demand (COD) and dye from liquorice extraction plant (LEP) wastewater. The addition of n-TiO_2_ improved the membrane hydrophilicity, the permeate flux values, and the flux recovery ratio (FRR) due to the presence of amine, hydroxyl, and silica groups on the membrane surface. The membrane containing 1 wt% NP showed the best parameters, including high clean water flux (37.2 kg/m^2^·h) and FRR value (98%), permeability flux (25 kg/m^2^·h), COD removal (88%), and dye removal (98%) (at COD concentration 800 mg/L, pressure 5 bar, after 150 min) [128].

Modification of flat polyethersulfone membranes with TiO_2_ nanoparticles in the amount of 0.4 wt% based on the phase inversion method was also carried out by Arsuaga et al. [112]. The TiO_2_ nanoparticles introduced into the structure changed the morphology of the membrane to a more open and porous membrane with antifouling properties and improved long-term stability of the flow. Strong correlations between some physicochemical properties, such as porosity, hydrophilicity, and permeability of the modified membranes, with the spatial distribution of particles in the membrane structure were observed. The membrane contamination process was investigated using BSA and humic acids as model organic pollutants, concluding that particle distribution is a key parameter in sediment reduction [112]. Other examples of membrane modification with n-TiO_2_ to catalytically reduce fouling or to improve self-cleaning properties are shown in Table 5.

## 7. Membranes Containing Nanoparticles of Iron

Iron nanoparticles have many specific properties that are determined by the structure and composition of the compound and by the size of the nanoparticles. The same compound may exhibit different properties, e.g., magnetic, depending on the method used for its synthesis and on the shape and size of the nanoparticle grains. Therefore, in the case of iron nanoparticles and its compounds, it is possible to obtain a huge variety of materials necessary for use in wastewater-treatment and water-treatment processes, allowing for direction of the processes taking place. Depending on the method of synthesis, iron (III) nanoparticles may have grains of various sizes and shapes, including spherical, uneven, quasi cube, parallel hexahedron, irregular sphere, nanosheet, and hexagon shapes [136] and may thus possess ferromagnetic, catalytic, oxidizing, and sorption properties of varying intensities. It has also been found that iron oxide nanoparticles, including Fe_3_O_4_ and Fe_2_O_3_, have intrinsic enzyme-like activities and are now considered new enzyme mimetics, so-called nanozyme [137]. Due to its nature, also in the case of modification of materials used in the production of membranes with iron nanoparticles/iron compounds, it is also important to use the optimal amount of nanoparticles [7].

One of the purposes of using iron nanoparticles in the modification of polymer membranes is the removal of toxic metals from the aquatic environment, including Cu or Pb ions. In order to remove Cu (II) from the aquatic environment, Daraei et al. [138] created a nanocomposite polymer membrane based on polyethersulfone (PES) and polyaniline (PANI) polymers and nanoparticles of iron oxide (II,III) Fe_3_O_4_ with grain sizes in the range of 12–28 nm and a thickness of about 8 nm for a polyaniline coating. The material developed in this way was used to remove copper (II) ions. A membrane containing 0.1 wt% of nanoparticles was characterized by the highest ion rejection in the range of 85% for an aqueous solution of Cu(NO_3_)_2_ with a concentration of 20 mg/L and 75% of Cu (II) ions from a solution with a much lower concentration, i.e., 5 mg/L. The results obtained in the research showed that the adsorption mechanism was the dominant process, and the Redlich–Peterson isotherm was the most likely adsorption isotherm, which expressed a relatively complex adsorption mechanism. The conducted studies also showed that the prepared nanocomposite membrane ensures durability in the filtration process and excellent suitability for reuse [138].

A modification of the membranes with n-Fe_3_O_4_ in order to remove Cu(II) ions was also carried out by Ghaemi et al. [139]. First, they modified the surface of Fe_3_O_4_ nanoparticles by immobilizing silica, metformin, and amine. In the second stage, a PES nanofiltration membrane with a mixed matrix was prepared by depositing various concentrations of modified nanoparticles based on n-Fe_3_O_4_. Nanoparticles of Fe_3_O_4_ treated with trisodium citrate were monodisperse and had an average diameter of about 9.2 nm. As a result of modification, Fe_3_O_4_/SiO_2_-Met, –amine nanoparticles, and Fe_3_O_4_ silica-coated nanoparticles were characterized by a typical core-shell structure with average sizes of 40–45 nm, 35–40 nm, and 40 nm, respectively. The deposition of iron oxide nanoparticles caused changes in the average pore radius, the porosity and hydrophilicity of membranes, and the presence of nucleophilic functional groups on nanoparticles, which contributed to a significant increase in the pure water stream and the ability to remove copper. The highest degree of copper removal in the range of 92% was recorded for the membrane consisting of 0.1 wt% Fe_3_O_4_ nanoparticles coated with metformin modified silica. Moreover, the reusability was found for the membrane with the best performance after several cycles of use/regeneration using Ethylenediaminetetraacetic acid (EDTA) as the eluting agent [139].

Another example of membrane modification to remove toxic metals is the work of Gholami et al. [140], who used ferrosoferric oxide nanoparticles to modify polyvinyl chloride (PVC)–cellulose acetate (CA) membranes to remove lead from the aquatic environment. Nanofiltration membranes were prepared by the phase inversion method with different contents of Fe_3_O_4_ nanoparticles. The obtained results showed that the use of a membrane containing 0.01 wt% of nanoparticles did not change the lead ion removal characteristics compared to membranes without modification. Increasing the concentration of nanoparticles resulted in the formation of more channels in the membrane, which in turn weakened the mechanical strength of the membrane. Therefore, it was necessary to determine the optimal membrane composition allowing for a compromise between lead removal efficiency and mechanical strength. The tests carried out showed that the membrane containing 40 wt% showed the best results in terms of flow characteristics and lead rejection: CA and 0.1 wt% Fe_3_O_4_ nanoparticles with 60-nm grains [140].

Modification of the membranes with iron nanoparticles changes the hydrophilicity of the material, an example of which is the work of Fang et al. [141]. The iron-tannin-framework (ITF) complex was introduced to a poly(ethersulfone) (PES) casting solution as a hydrophilic additive to fabricate ITF/PES ultrafiltration (UF) membranes by using non-solvent-induced phase separation (NIPS). The obtained results show that ITF can regulate the porous structure and surface properties of PES membranes. It was found, inter alia, that compared to classic PES membranes, ITF/PES membranes have increased hydrophilicity and porosity as well as a reduced size of surface pores while increasing the permeability and separation efficiency. It should be noted, however, that the porosity of the membrane first increased and then slightly decreased with increasing ITF content from 0.15 to 0.9 wt%, while the average pore size decreased from 16.1 to 13.7 nm with increasing load of ITF. The modified membranes also showed better fouling resistance and stable hydrophilicity, which remained constant for 30 days from the moment of incubation in deionized water [141]. 

The diverse magnetic properties of iron nanoparticles and its compounds enable the targeting of processes and reduction of selected pollutants as well as the possibility of an effective separation and isolation process of selected substances, including the removal of viruses (Table 6).

Some iron nanoparticles have negative properties towards microorganisms. They limit the transport of nutrients to bacterial cells, causing nutritional imbalance and metabolic weakening of bacteria, and they generate oxidative stress by producing some reactive oxygen species (ROS) with free radicals (Fenton or Fenton-like reaction), damaging cellular proteins, lipids, and DNA and eventually leading to bacterial death [149]. However, it should be remembered that, for some microorganisms, iron may be a factor necessary for their development and multiplication; therefore, depending on the composition of the biocenosis of the purified solution, both the composition of nanoparticles as well as their size and shape must be properly adjusted.

## 8. Membranes Containing Nanoparticles of Other Nanoparticles of Metals

Metal nanoparticles such as Cu, Zn, Mg, Mn, Se, and Ni with various oxidation states, structures, and various organic and inorganic systems are used to modify polymer membranes. The applicability depends on the purpose of the modification, cost-consumption, and effectiveness of subsequent processes removing pollutants from the water environment. They can improve the properties of modified materials as well as direct the processes taking place in the environment being treated. The biocidal property is an important feature taken into account. Therefore, one of the metals used to modify polymer membranes are copper and selenium. It should be noted, however, that nanoparticles, depending on the composition, shape, and size of the grain, are characterized by various biocidal properties. For example, copper oxide nanoparticles show greater biocidal properties than metallic copper nanoparticles. Copper oxide nanoparticles release copper ions as a result of dissolution. In addition, the redox cycle between Cu (I) and Cu (II) ions generates reactive peroxide species that contribute to the degradation of biomolecules. In the case of metallic copper nanoparticles, the mechanism of biocidal action is based on the production of larger amounts of ROS, which leads to a number of interactions at the level of structural proteins, organelles, and DNA, contributing to oxidative stress in the cell [149]. In addition, copper nanoparticles, similar to selenium nanoparticles, show very good antioxidant activity, which allows them to be widely used in water purification and treatment processes. Akar et al. [150] used Se and Cu nanoparticles with grains ranging from 90 in the case of n-Cu to even 175 nm in the case of n-Se to modify the polyethersulfone ultrafiltration membrane by using the phase inversion process. The tests included membranes with four different weight ratios of n-Se and n-Cu to PES: 0.002, 0.010, 0.030, and 0.050. The antifouling properties were determined using the activated sludge as a biological suspension, while the protein rejection potential was performed using a bovine serum albumin (BSA) solution. The obtained results indicate that, in comparison with the pure PES membrane, the 0.05 Cu/PES membrane is characterized by the highest (86.3%) protein rejection ratio while the Se/PES membranes showed a better antifouling effect [150].

The use of nanoparticles should be verified for possible leaching and release into the solution, which can affect water contamination. For example, Kar et al. [151] observed that copper nanoparticles leach from the host polymer matrix (i.e., polysulfone or N-methylpyrrolidone) into the gelling medium when present alone or with silver [151]. 

The biocidal properties of Cu nanoparticles were also used in the work of Ben-Sasson et al. [152]. They carried out a modification on the surface of a thin-film composite reverse osmosis membrane. It was found that the membrane modified with Cu-NP shows a strong antibacterial effect, leading to a reduction in the number of live *Escherichia coli* bacteria by 90% more than the unmodified membrane, which in turn leads to a reduction of biofouling [152]. Similar results were obtained by Zhang et al. [153]. Antifouling tests were carried out on membranes made of a thin-layer polyamide- carboxylated chitosan composite (PA-CCTS) modified with copper nanoparticles, achieving an antibacterial effectiveness of over 99% and durability of up to 90 days from the moment of immersion in water. In addition to the possibility of reducing the amount of bio-sediment, they also found the release of Cu^2+^ ions in a slow and sustained manner due to the helix effect between CCTS and Cu^2+^. PA-CCTS-Cu also showed better anti-protein properties of the unmodified membrane and higher recovery after cleaning in a soil test using bovine serum albumin sediment as a model soil contaminant. The improvement in some properties was attributed to the increased hydrophilicity of the modified membrane [153].

Fouling processes make it difficult to carry out treatment processes and to reduce efficiency, therefore, many targeted works aimed at minimizing the impact of this process on water and wastewater treatment. To minimize the fouling process, Liang et al. [154] used ZnO nanoparticles to modify PVDF based on the wet phase separation method. ZnO-NP was added to the membrane matrix in various proportions to modify the inner pore surfaces of the membrane. All modified membranes achieved almost 100% water stream recovery after physical purification, while the crude membrane achieved only 78%. Taking into account the other elements affecting the effectiveness of the membrane, including mechanical strength, it was found that the most optimal dose of ZnO nanoparticles is 6.7 wt% [154]. Jo et al. [155] had high antifouling efficiency along with bactericidal properties. Ultrafiltration membrane with antibacterial effectiveness and high water flux, they prepared from polyethersulfone (PES) and zinc oxide nanoparticles grafted with poly (1-vinylpyrrolidone) and poly (1-vinylpyrrolidone-co-acrylonitrile) [155]. 

Modification of the membrane based on ZnO nanoparticles allows for an improvement in hydrophilicity and performance in terms of permeability, porosity, and rejection ability, as exemplified by the modification of membranes such as PSF [156,157], PVDF microfiltration [158,159,160], PES [161,162], as well as PS [163]. Performed by Zhang et al. [149], the research showed that polyvinylidene fluoride membranes modified with ZnO nanoparticles prepared by two different methods were characterized by higher hydrophilicity, permeability, and antifouling effect. Moreover, the developed hybrid membranes showed better adsorption and desorption properties of copper ions [164].

An interesting solution in developing the possibility of modifying membranes is the possibility of applying the response surface methodology (RSM), used to calculate complex interactions between independent process parameters. Ahmad et al. [165] applied this method to determine the possibility of modifying a polyethersulfone (PES) and polyvinylpyrrolidone (PVP) membrane with ZnO by the phase inversion technique. The purpose of the modifications was to be able to use such modified membranes to remove humic acid. The synthesis parameters included weight percent PES, ZnO-NP, PVP, and the evaporation time of the solvent. Models were developed that allowed for the correlation of different membrane compositions with three indicators characterizing the membrane performance, such as the pure water flux (PWF), humic acid flux (HAF), and humic acid rejection (HAR). On the basis of the modeling, it was shown that the most optimal conditions for membrane preparation were 17.25, 3.62, and 3.75 wt% and 15 s for PES, ZnO-NP, PVP weight percent, and solvent evaporation time, respectively. The membrane thus obtained allows to obtain rejection of humic acid even at the level of 96.34% [165].

An interesting solution is membranes modified with nanoparticles of complex compounds, such as the boron-doped TiO_2_-SiO_2_ cobalt ferrite (B-TiO_2_-SiO_2_/CoFe_2_O_4_) nanophotocatalyst using the phase inversion technique [166]. The addition of nanoparticles improved the porosity, morphology, structure, pure water flux, antifouling properties, and separation efficiency of the embedded membranes thanks to the hydrophilic and photocatalytic properties of the boron nanoparticle and Ti, Si, and Co-Fe oxides. A membrane containing 0.5 wt% nanoparticles has the highest clean water stream and water stream recovery rate with the best separation parameters [166]. In order to improve the performance of the membrane and its antifouling properties, the L-Histidine-doped polyethersulfone (PES) membrane was modified with the TiO_2_-CdS nanocatalytic nanocomposite. The addition of the L-Histidine-doped nanocomposite increased the porosity of the membrane and its hydrophilic properties and membrane permeability. The obtained results showed that the membrane containing 0.5 wt% NPs allows to obtain the optimal effects of pollutant removal, including the removal of COD at the level of almost 100% during the filtration of biologically treated sewage from a palm oil mill (COD concentration of 1000 mg/L at a flow rate of 150 L/h and a pressure of 5 bar). It has been found that increasing the feed concentration has a negative effect on the membrane performance, while the feed flow rate increases the separation efficiency and the self-cleaning properties of the membrane [167].

Modification of the membranes is carried out for a specific purpose, e.g., for textile and dyeing wastewater treatment processes. To this end, Yu et al. [168] developed a conductive polyvinylidene fluoride (PVDF) membrane modified with Ni nanoparticles. Moreover, the membrane was connected to an external electric field, which allowed for adequate aeration (aeration in situ). The PVDF-Ni membrane showed 94% flux recovery after filtration of Bovine Serum Albumin (BSA) and showed a clear resistance to dye uptake and antibacterial activity. These versatile properties combined with an easy production process showed great prospects for the application of the PVDF-Ni membrane in the treatment of textile and dyeing wastewater [168].

Detailed examples of the use of different types of selected metals in nanomembranes are presented in Table 7.

## 9. Conclusions

The huge variety of metal nanoparticles and metal compound nanoparticles makes it possible to use them to develop polymer membranes with specific parameters. For modification of membranes, not only nanoparticles with biocidal, magnetic, and strong oxidizing properties but also those that, when combined with the membrane, will improve mechanical, antifouling, and durability are used. Silver, iron, zirconium, silica, aluminum, titanium, and zinc nanoparticles are widely used, both as pure metals and in the form of oxides or bimetals. Since polymer membranes are characterized by hydrophobic properties, which often makes it difficult to use in an aqueous environment, the addition of metal nanoparticles allows for surface modification and for obtaining a material with strong hydrophilic properties. Various methods are used in the modification processes, including phase inversion, proprietary methods using mixing, ultrasound, drying, and heating. Each method allows to obtain an optimal material that can be used in processes removing pollutants from the aquatic environment, including the removal of toxic metals, bacteria and viruses, and organic compounds. However, it should be remembered that increasing the amount of nanoparticles used for modification does not always lead to an optimal material. Many times, a compromise has to be made between improving physical parameters and the efficiency of cleaning solutions.

## Figures and Tables

**Table 1:** Membrane water-purification processes for potential use in water purification in hazardous areas.

Process Type	Principle of Operation	Advantages/Disadvantages	Ref.
**Pressure processes**			
Microfiltration	It is used mainly in ceramic filters or polymer membranes.	The main disadvantage is the low efficiency, and in order to increase it, a support is used, for example, a sand filter. The disadvantage of using ceramic membranes is the possibility of releasing arsenic from contaminated sintered materials.	[11,12]
Ultrafiltration	The use of a polymer ultrafiltration membrane enables good purification in one operation.	Microbiologically safe water is easily produced.	[13]
Nanofiltration	Nanomembranes remove polyvalent ions and organic compounds.	They cannot be used as the only solution for the desalination of sea water.	[14]
Reverse osmosis	It takes place in a solution with a higher concentration of the solute to a solution with a lower concentration, i.e., it leads to an increase in the concentration differences of both solutions.	Very good results are obtained in the removal of radionuclides, used to treat water after the 2011 Fukushima earthquake/very high energy consumption.	[15]
**Osmotic processes**			
Spontaneous osmosis	It uses a two-layer filter with an inner layer containing a semipermeable osmotic layer, e.g., concentrated sugar solution.	Regeneration is achieved by exchanging the osmotic layer. It removes a wide range of contaminants without the use of pressure.	[16]
**Thermal processes**			
Membrane distillation	It is a hybrid process: water evaporation, water vapor filtration through a membrane, and water vapor condensation.	The process can be carried out at a temperature of 50–90 °C and works well in regions with high sun exposure.	[17]

**Table 2:** Selected applications of various types of silver in nanomembranes.

NP	Polymer	Particle Size/Loading, wt%	Fabrication Method	Application/Performance	Ref.
Ag-GO	Polyamide 6,6	0.8, 1.0 wt%	Graphene oxide was synthesized by using Hummers’ method/wet phase inversion method.	135% flux increase, 40% less irreversible fouling, 46% increase in hydrophilicity, and excellent antibacterial properties against *E. coli*	[89]
Ag	PES	10 nm	Acrylamide was grafted onto a PES membrane surface, and Ag nanoparticles were formed within the grafted layer.	positive effect on hydrophilicity, antifouling properties, and excellent antibacterial properties against *E. coli* (99.99%)	[90]
Ag	PSU	30–70 nm/2–4 wt%	Phase inversion via immersion precipitation technique/4 wt% of PVP was used as a pore former in the casting solution.	UF/positive effect on hydrophilicity, antifouling properties, BSA rejection, and excellent antibacterial properties against *E. coli*	[91]
Ag	PES	<100 nm	NIPS process using PVP as an additive and N-Methyl-2-pyrrolidone as a solvent	superb antibacterial and anti-biofouling performances	[92]
Ag	PES	25–50 nm	Own modification method consisting in soaking the PES hollow fiber membrane in a solution of silver ions, diffusion of ions into the polymer, and their reduction with ascorbic acid	positive effect on hydrophilicity and lower biofouling tendency (about 15% higher permeability)	[93]
Ag	PVDF	<100 nm	PEG as a hydrophilic agent, zeolitic-like framework-67 (ZIF-67), ethylenediamine as a cross-linking agent on n-Ag-decorated polyester textile support	good wettability, high pure water flux (PWF; 35.8 L/m^2^⋅h), flux recovery ratio (FRR; 90%), and dye removal efficiency (96.41%)	[94]

**Table 3:** Selected applications of various types of silicas in nanomembranes.

NP	Polymer	Particle Size/Loading, wt%	Fabrication Method	Application/Performance	Ref.
Silica (LUDOXs HS-40)	PA	~13.2 nm/5–28% of PA	IP	Filtration of dioxane solution/positive effect on Pw; negative impact on solute rejection	[97]
Nanosilica	PA	3 and 16 nm/0–0.4% (3 nm) and 0–0.5% (16 nm) in aqueous phase	IP	Reverse osmosis RO/positive effect on Pw and thermal stability; negative impact on NaCl rejection	[98]
Functionalized Silica	PA	0.04–0.4% in aqueous phase	IP	RO; pervaporation PV/positive effect on thermal stability and Pw; negative impact on NaCl rejection	[99]
Mesoporous silica (MCM-41) and nonporous silica	PA	Both ~100 nm/0–0.1% in organic phase (0.05%)	IP	RO/positive effect on surface hydrophilicity and Pw; salt rejection no change porous; structures of filler contributed significantly	[100]
Modified mesoporous silica	PA	~100 nm/0–0.07% in aqueous phase (0.03%)	IP	NF/under 87 psi, optimal water flux is 32.4 L/m^2^ h; Na_2_SO_4_ rejection (4 80%, 5 mmol/L)	[101]
Silica	Fluoropolyamide	0–1.0% (*w*/*v*) in aqueous phase (0.1)	IP	NF/positive effect on Pw; Na_2_SO_4_ rejection under 87 psi; optimal water flux is 15.2 L/m^2^ h; Na_2_SO_4_ rejection (85.0%, 2000 mg/L)	[102]
L-cysteine modified-POSS nanoparticles	Polyetherimide	33.77 nm/0.001–1 wt%	IP	NF/positive effect on salt rejection; the best separation performance for membrane with 1 wt% L-cysteine modified-POSS nanoparticles	[103]

**Table 4:** Select applications of various types of aluminum in nanomembranes.

NP	Polymer	Particle Size/Loading, wt%	Fabrication Method	Application/Performance	Ref.
γ-Al_2_O_3_	PVDF	20 nm/1–4 wt%	One-step conventional phase inversion technique	UF/solution of BSA/PBS (1 g/L, pH = 7.4)/positive effect on separation performances	[110]
Al_2_O_3_	PVDF	10 nm/2 wt%	Phase inversion technique	UF/oil–wastewater ultrafiltration/positive effect on surface hydrophilicity	[111]
Al_2_O_3_	PES	80 nm/0.4 wt%	Phase inversion technique	UF/BSA and humic acids as model organic foulants/more open and porous structure and antifouling property, and better long-term flux stability	[112]
γ-Al_2_O_3_	PSF	30 nm/0.02 and 0.03 wt%	Blending method and phase inversion technique	UF/bioreactors/prevents biofilm formation and reduces resistance by 75%	[113]
Al_2_O_3_	PA	14 nm/1 wt%	In situ interfacial polymerization	OM/positive effect on permeate flow and hydrophilicity, maintaining salt rejection	[114]
Al_2_O_3_	PES HF/PEO-PPO-PEO	3.6, 7.4, and 10.9 wt%	In situ vapor-induced hydrolyzation	UF/antifouling properties to humic acid	[115]

**Table 5:** Select applications of various types of titanium in nanomembranes.

NPs	Polymer	Particle Size/Loading, wt%	Fabrication Method	Application/Performance	Ref.
TiO_2_/SiO_2_	PEI/PAN	30, 45, 90, and 110 nm/0.2, 0.4, 0.6, and 0.8 wt%	mineralization, ultrasonic mixing, coating, casting, and drying	SRNF/organic solvents (n-heptane, toluene, butanone, ethyl acetate, isopropanol, and polyethylene glycol)	[129]
TiO_2_	PVDF–PEG	20–30 nm/0.25 to 2.0 wt%	the phase inversion process (PIP) method	UF/photocatalytic NOM degradation/good self-cleaning ability	[130]
TiO_2_	PVDF	16 nm/7.5 wt%	mixing, cooling, degassing with ultrasound, and spinning	UF/photocatalytic nonylphenol degradation	[131]
TiO_2_	PPMMs	50–100 nm/2.9–12.9 wt%	photoinduced reversible addition-fragmentation chain transfer grafting polymerization of acrylic acid	SMPR/phenol decomposition	[132]
TiO_2_	PAA/PVDF	0.5, 1.5, and 3.0%, m/v	plasma treatment at 100 W for 120 s followed by liquid grafting with 70% aqueous AA at 60 °C for 2 h	the highest pure water flux and the best protein antifouling property; photodegradation of strongly bound foulants; removal of 30–42% of 50 mg/L aqueous Reactive Black 5 (RB5) dye	[133]
GO-TiO_2_	PSF/PVP	0–5 wt%	in situ sol-gel	UF/humic acids removal	[134]
TiO_2_	nonwoven fabric-reinforced PSF	10 nm	interfacial polymerization of MPD in the aqueous phase (2 wt%) and TMC in the organic phase (0.1 wt%)	RO/photobactericidal effect on *Escherichia coli*	[135]

**Table 6:** Selected applications of various types of iron in nanomembranes.

NPs	Polymer	Particle Size/Loading, wt%	Fabrication Method	Application/Performance	Ref.
γ-Fe_2_O_3_	PSF	10 nm/0.1–0.9 wt%	IP of thin film nanocomposite (TFNC)	RO/better hydrophilicity/high NaCl salt rejection of 98%	[142]
Fe_2_O_3_	PVP	<50 nm/0–2 wt% (the most optimal: 1 wt%)	the phase inversion method	UF/positive effect on hardness, hydrophilicity, sodium alginate (SA) rejection rate (91.9%), and antifouling properties	[143]
ZVI	PSF/PVP	100 nm (change due to the oxidation of the ZVI)	the phase inversion method	UF/oxidation of ZVI nanoparticles to FeO(OH), which are mechanically stable	[144]
FeO	PES	20 nm/1–4 wt%	the phase inversion method	UF/positive effect on hydrophilicity, thermal stability, and dye rejection	[145]
Fe_3_O_4_	PSF	<50 nm/3.9 wt%	membranes were coated by a simple filtration protocol	Low-pressure membrane systems/removal of a model virus (bacteriophage MS2) with efficiency exceeding 99.99%	[146]
Pd/Fe	MMMs from CA	10 nm of Fe and 11–30 nm of Pd/Fe	magnetic stirring, dispersion, drying, and liquid ethanol bath	UF/de-chlorination of TCE	[147]
Fe/Ni	PVDF, Nylon-66, Millex GS, mixed cellulose ester membranes	56 nm in nylon-66, 82 nm in PVDF, 43 nm in MCEM, and 36 nm in Millex G	immersion of membranes in coating solutions and heating	MF/degradation of chlorinated (PCE, TCE, CT, and dichloromethane) and non-chlorinated hydrocarbons (methane and ethane)	[148]
Fe/Fe_2_O_3_	PA-PSF	<30 nm/0.25 wt%	interfacial polymerization process (PI)	RO/positive effect on roughness and bactericidal effect	[149]

**Table 7:** Select applications of various types of selected metals in nanomembranes.

NP	Polymer	Particle Size/Loading, wt%	Fabrication Method	Application/Performance	Ref.
ZrO_2_	PES	<100 nm/0.01, 0.03, 0.05, 0.07, and 0.1 ZrO_2_/PES ratios (w/w)	Phase inversion technique	MBR/activated sludge filtration/positive effect on strength, permeability, and antifouling properties	[169]
ZrO_2_	PVDF	<100 nm/20 wt%	Casting and immersion into a water bath of ternary suspensions	UF/positive effect on hydrophilicity and antifouling properties	[170]
Au	PES	39 nm/0.1 wt%	Phase inversion technique	NF/for removing PO_4_^3−^/positive effect on hydrophilicity and antifouling properties	[171]
Mg(OH)_2_	PVDF	<100 nm/10 wt%	Phase inversion technique	positive effect on strength, permeability, antifouling properties, BSA rejection, and antibacterial properties against *E. coli*	[172]
MgO	PPSU	55–105 nm/0.25 wt%	Phase inversion technique	improvement in membrane properties such as interlayer spacing, hydrophilicity, flux, rejection, porosity, pore size, and oleophobicity relative to oil/water emulsions	[173]
CuO	PA	<100 nm	Low-temperature metallic (metallic-gas) plasma and nonmetallic (gas) plasma of the physical vapor deposition process	high performance of the filtration materials with a strong antibacterial activity against gram-negative bacteria (*E. coli*)	[174]
Cu/TNT	PES	TNT: 50–200 nm, Cu: 2–3 nm (on the outer surface of the TNTs)	Wet phase inversion technique	positive effect on permeability, BSA rejection, and antibacterial properties against *E. coli* (lower in relation to *S. epidermidis*)	[175]

## Abbreviations

ALD	Atomic layer deposition
BSA	Bovine serum albumin
CA	Cellulose acetate
CPTES	3-cyanopropyltriethoxysilane
CT	Carbon tetrachloride
Cu/TNTs	Titanate nanotubes modified with copper
EDTA	Ethylenediaminetetraacetic acid
EIPS	Evaporation-induced phase inversion
GO	Graphene oxide
HA	Humic acid
HAF	Humic acid flux
HAR	Humic acid rejection
HF	Hollow fiber
IP	Interfacial polymerization
ITF	Iron-tannin framework
MBR	Membrane bioreactor
MF	Microfiltration
MMMs	Mixed matrix membranes
MMNM	Nanocomposite mixed matrix membrane
MPD	m-phenylenediamine
NF	Nanofiltration
NIPS	Non-solvent-induced phase inversion
NOM	Natural organic matter
OM	Osmosis membranes
OSN	Organic solvent nanofiltration
PA	Polyamide
PAA	Poly(acrylic acid)
PA-CCTS	Polyamide-carboxylated chitosan composite
PAN	Polyacrylonitrile
PANI	Polyaniline
PBS	Phosphate-buffered saline
PCE	Tetrachloroethylene
PEG	Polyethylene glycol
PEI	Polyethyleneimine
PEMEC	Proton exchange membrane fuel cells
PEO-PPO-PEO	Poly(ethylene glycol)-block-poly(propylene glycol)-block-poly(ethylene glycol)
PES	Polyethersulfone
PI	Polyimide
Polyamide 6,6	Poly(hexamethylene adipamide)
POSS	Polysilsesquioxanes
PP	Polypropylene
PPMMs	Polypropylene macroporous membranes
PPSU	Polyphenylsulfone
PSF	Polysulfone
PTEE	Polytetrafluoroethylene
PVB	Polyvinylbutyral
PVC	Polyvinyl chloride
PVDF	Polyvinylidene fluoride
PVP	Polyvinylpyrrolidone
Pw	Water permeability
PWF	Pure water flux
RO	Reverse osmosis
ROS	Reactive oxygen species
RSM	Response surface methodology
SIS	Sequential infiltration synthesis
SMPR	Submerged membrane photoreactor
SPES	Sulfonated polyethersulfone
SRNF	Solvent-resistant nanofiltration
TA	Tannic acid
TCE	Trichloroethylene
TFN	Thin-layer nanocomposite
TFNC	Thin-film nanocomposite
TIPS	Thermally induced phase inversion
TMC	Trimesoylchloride
UF	Ultrafiltration
VIPS	Vapor-induced phase inversion
ZVI	Zero valent iron nanoparticles
